# The Optimization of Assay Conditions and Characterization of the Succinic Semialdehyde Dehydrogenase Enzyme of Germinated Tartary Buckwheat

**DOI:** 10.3390/foods13010017

**Published:** 2023-12-20

**Authors:** Yuchan Yang, Jiashang Liu, Nan Li, Yu Guo, Hua Ye, Zhanming Li, Dongxu Wang, Yuanxin Guo

**Affiliations:** 1 School of Grain Science and Technology, Jiangsu University of Science and Technology, Zhenjiang 212100, China; 221211803113@stu.just.edu.cn (Y.Y.); 222241821504@stu.just.edu.cn (N.L.); gy18234591259@163.com (Y.G.); huaye@just.edu.cn (H.Y.); lizhanming@just.edu.cn (Z.L.); wdx@just.edu.cn (D.W.); 2 Catering and Food Department, Inner Mongolia Vocational College of Commerce, Hohhot 010070, China; liujiashang2022@163.com

**Keywords:** germinated Tartary buckwheat, succinic semialdehyde dehydrogenase, enzyme activity assay, enzymatic characteristics, response surface analysis

## Abstract

In this study, the conditions for optimizing the determination of succinic semialdehyde dehydrogenase (SSADH, EC 1.2.1.79) activity in germinated Tartary buckwheat were investigated. Based on a single-factor test, the effects of temperature, pH, and succinic semialdehyde (SSA) concentration on the enzyme activity of germinated buckwheat SSADH were investigated by using the response surface method, and optimal conditions were used to study the enzymatic properties of germinated buckwheat SSADH. The results revealed that the optimum conditions for determining SSADH enzyme activity are as follows: temperature—30.8 °C, pH—8.7, and SSA concentration—0.3 mmol/L. Under these conditions, SSADH enzyme activity was measured as 346 ± 9.61 nmol/min. Furthermore, the thermal stability of SSADH was found to be superior at 25 °C, and its pH stability remained comparable at pH levels of 7.6, 8.1, and 8.6 in germinated Tartary buckwheat samples; however, a decline in stability was observed at pH 9.1. Cu^2+^, Co^2+^, and Ni^2+^ exhibited an activating effect on SSADH activity in germinating Tartary buckwheat, with Cu^2+^ having the greatest influence (*p* < 0.05), which was 1.21 times higher than that of the control group. Zn^2+^, Mn^2+^, and Na^+^ inhibited SSADH activity in germinating Tartary buckwheat, with Zn^2+^ showing the strongest inhibitory effect (*p* < 0.05). On the other hand, the K_m_ and V_max_ of SSADH for SSA in germinated Tartary buckwheat were 0.24 mmol/L and 583.24 nmol/min. The Km and Vmax of SSADH for NAD^+^ in germinated Tartary buckwheat were 0.64 mmol/L and 454.55 nmol/min.

## 1. Introduction

Tartary buckwheat, which is a dicotyledonous plant of the genus Fagopyrum of the family Polygonaceous, is the traditional dominant grain in China and is widely planted in northwest and southwest China [1]. Tartary buckwheat not only has very high nutritional and medicinal value but it also has strong adaptability to the environment [2]. Tartary buckwheat is rich in flavonoids such as rutin, which can regulate and improve the endocrine system and has anti-hypertension [3], anti-oxidation [4], and anti-hyperglycemia functions [5]. Germination is an important reason for the enrichment of the functional components of Tartary buckwheat, such as flavonoids, resistant starches, and γ-amino butyric acid (GABA) [6]. Hao et al. [7] utilized a treatment involving slightly acidic electrolyzed water to promote the germination of buckwheat, and the findings of this study revealed that this treatment effectively enhanced the accumulation of GABA and rutin in germinated buckwheat. At present, the existing research on germinated Tartary buckwheat mainly focuses on the properties and changes of flavonoids [8]. However, there has been little discussion about the enrichment of GABA in germinated Tartary buckwheat.

GABA is a non-protein amino acid that participates in a variety of metabolic activities in vivo. It is an important inhibitory neurotransmitter which has physiological effects such as activating the liver, regulating hormones, and calming nerves. In addition, it plays an important role in preventing cardiovascular disease [9]. In higher plants, the GABA shunt serves as the primary pathway for the synthesis and conversion of GABA. This process is predominantly facilitated by the enzymes glutamate decarboxylase (GAD), GABA aminotransferase (GABA-T), and SSADH. GAD, as a crucial enzyme in GABA synthesis, plays a pivotal role in catalyzing the production of GABA. Conversely, GABA-T and SSADH act as rate-limiting enzymes in GABA degradation, contributing to the breakdown of GABA [10]. In the GABA shunt [11], the enzyme glutamate decarboxylase (GAD) facilitates the conversion of L-glutamic acid (L-Glu) through α-decarboxylation to produce γ-aminobutyric acid (GABA). Subsequently, GABA transaminase (GABA-T) catalyzes the conversion of GABA to succinic semialdehyde (SSA). SSA is further metabolized into succinic acid through the action of succinic semialdehyde dehydrogenase (SSADH), subsequently entering the tricarboxylic acid (TCA) cycle. Notably, the reaction catalyzed by GABA-T is reversible, while the reaction catalyzed by SSADH is irreversible. The GABA pathway exhibits the potential to enhance the organism’s ability to respond to both biotic and abiotic stresses [12].

SSADHs are widely distributed across various organisms, including humans, bacteria, plants, and mammals. In humans, a mutation or deficiency in SSADH leads to the elevation of GABA levels within the body, resulting in the manifestation of a rare genetic disorder [13]. Analogous deficiencies in plants result in diverse developmental and phenotypic alterations [14]. In plants, SSADH facilitates the final step of the GABA metabolic pathway by employing NAD+ as a coenzyme to irreversibly oxidize SSA into succinic acid. SSADH is the rate-limiting enzyme of GABA metabolism, which is found in the mitochondria [12,15,16] and plays an important role in the enrichment of GABA. In the GABA shunt, GABA is enzymatically converted to SSA by GABA-T in a reversible reaction. Subsequently, SSA is further metabolized to succinic acid through the catalytic action of SSADH. If the activity of SSADH enzyme is diminished, its ability to convert SSA into succinic acid will be compromised, leading to the intracellular accumulation of SSA and, consequently, an elevation in GABA levels [17]. Sam et al. [18] combined two genetic approaches to suppress the severe phenotype of SSADH mutants as a way to elucidate the role of succinic semialdehyde (SSA), C-hydroxybutyrate (GHB), and GABA in reactive oxygen intermediate accumulation. In addition, appropriate concentrations of sodium chloride can increase the expression of SSADH in Arabidopsis, thereby affecting the overall metabolism of GABA [19].

Various germinating methods have been devised to enrich the GABA of seeds. For example, germinated soybean and buckwheat have been reported to exhibit GABA levels ranging from 0.50- to 2.60-fold and 0.60- to 37.5-fold higher than their respective seeds [20,21]. Moreover, the antagonistic effect of a 30 w ultrasound treatment and 2.0% CaCl_2_ stress with the germination of brown glutinous rice for 9 h resulted in a substantial, 3.29-fold increase in GABA contents [22]. Furthermore, the germination treatment exerted an influence on the enzymatic activity within the grain. Xu et al. [23] discovered that the accumulation of GABA in soybean during germination primarily resulted from alterations in GAD activity, which also exhibited a correlation with GABA-T activity. Tartary buckwheat is widely considered to be a functional health food that is rich in flavonoids and GABA. The existing research on the enrichment of GABA in Tartary buckwheat at home and abroad mainly focuses on changing the germination conditions of Tartary buckwheat, such as germination [7], hypoxia combined with salt stress [24,25], aeration treatment [26], etc. However, there is limited research on the key enzymes involved in the enrichment process of Tartary Buckwheat, particularly regarding SSADH. In this study, the enzyme activity of germinated buckwheat SSADH was optimized, and its enzymatic properties were investigated. These findings are expected to serve as a foundation for the further analysis of the GABA-enriched mechanism in germinated buckwheat SSADH and provide crucial technical support for the future commercialization of GABA-rich buckwheat foods in China.

## 2. Materials and Methods

### 2.1. Materials and Reagents

The type of Tartary buckwheat seeds used in the experiment were Chuanqiao No. 1 seeds, produced in Liangshan, Sichuan. Semialdehyde succinate (SSA) and nicotinamide adenine dinucleotide (NAD^+^) were provided by Shanghai Yuanye Biotechnology Co., Ltd. (Shanghai, China), and the other reagents (analytical grade) and standards were purchased from Sinopharm Chemical Reagent (Shanghai, China).

### 2.2. Tartary Buckwheat Germination Test

The Tartary buckwheat seeds (20 g) were washed with deionized water, disinfected with 1% sodium hypochlorite solution for 15 min, washed with deionized water to neutral pH, and finally soaked in deionized water at 30 °C for 4 h. Next, Tartary buckwheat seeds were placed in a Petri dish with two layers of filter paper and placed in a biochemical incubator (HWS-150, Shanghai Senxin Experimental Instrument Co., Ltd., Shanghai, China) for dark germination at 31 °C. The germination humidity was about 85~90%. Meanwhile, deionized water was sprayed every 8 h to keep the Petri dish moist, and the germination time was 5 days [24].

### 2.3. SSADH Extraction

The dehulled germinated Tartary buckwheat (20 g) was placed in an ice bath, followed by the addition of 75 mL extract (1/15 mol/L PBS buffer, pH 8.6, 1 mmol/L EDTA, 20 mmol/L β-mercaptoethanol, 1 mmol/L PMSF), and a small amount of quartz sand was also added. The seeds were ground into homogenate under ice bath conditions and filtered with three layers of gauze. Finally, the homogenate was centrifuged at 1000 rmp and 4 °C for 20 min using a high-speed frozen centrifuge (KDC-160HR Keda Innovation Co., Ltd., LTD. Zhongjia branch, Hefei, China). The resulting supernatant represented the extract obtained from germinated Tartary buckwheat SSADH.

### 2.4. Fractional Precipitation of SSADH

The SSADH extract of germinated Tartary buckwheat was divided into 10 parts, 4 mL for each part. The SSADH in the extract was precipitated with different saturations of (NH_4_)_2_SO_4_ (0%, 20%, 30%, 40%, 5%, 60%, 70%, 80%, 90%, 100%). After being left to stand at 4 °C for 1 h and centrifuged at 10,000 rmp and 4 °C for 15 min, the supernatant was taken to determine the residual enzyme activity of SSADH, and the optimum saturation of SSADH in germinated Tartary buckwheat was determined.

The optimal saturation range of (NH_4_)_2_SO_4_ (X1%–X2%) was determined through the experiments. Then, X1% of (NH_4_)_2_SO_4_ was added to the extract of germinating Tartary buckwheat containing SSADH. The resulting supernatant was collected and saturated with (NH_4_)_2_SO_4_ at X2%. These procedures were repeated, and the precipitate was subsequently collected. To obtain the SSADH crude enzyme solution, the precipitate was dissolved in an equal volume of (1/15 mol/L) PBS (pH 8.6, 1 mmol/L EDTA, 20 mmol/L β-mercaptoethanol, 1 mmol/L PMSF).

### 2.5. Optimization of SSADH Enzyme Activity Determination Conditions

Single-factor experiment of SSADH activity assay: The 200 uL reaction system consisted of 0.2 mmol/LSSA, 2 mmol/L NAD^+^, 1 mmol/L EDTA, 1 mmol/L PMSF, 1/15 mol/L PBS buffer (pH 8.6), 20mmol/L β-mercapto ethanol, and 16 μL of crude enzyme solution. The absorbance value (722E Visible Spectrophotometer Shanghai Spectrometer Co., Ltd., Shanghai, China) of the crude SSADH enzyme solution from germinated Tartary buckwheat was measured at a reaction temperature of 30 °C within a time frame of 10 min. The optimal conditions for the three single factors of enzymatic reaction temperature, buffer pH, and SSA substrate concentration were optimized. The single-factor experimental conditions were as follows: the reaction temperatures were 20, 25, 30, 35, 40, and 45 °C; the substrate concentrations were 0, 0.1, 0.2, 0.3, 0.4, and 0.5 mmol/L. The pH values of the buffer solution were 0, 0.1, 0.2, 0.3, 0.4, and 0.5 mmol/L.

Based on the single-factor experiment, the three factors of temperature (A), pH value (B), and SSA concentration (C) were selected to design the response surface test (Table 1). The quadratic polynomial equation between the response value (Y) and the variables (A, B, C) was optimized, with the SSADH activity (Y) of germinated Tartary buckwheat serving as the response value.

### 2.6. Optimization of SSADH Enzyme Activity Determination Conditions

#### 2.6.1. Stability Analysis of SSADH

(1) Thermal stability: The crude enzyme solution of germinated buckwheat SSADH was kept at 25 °C, 30 °C, and 35 °C, and the residual enzyme activity was measured every 30 min. (2) pH stability: The germinated Tartary buckwheat SSADH crude enzyme solution was kept at different pH (pH 7.6, pH 8.1, pH 8.6, and pH 9.1), and the residual enzyme activity was measured every 30 min.

#### 2.6.2. Effects of Different Metal Ions on SSADH

The reaction system was added with 2 mmol/L metal ions to determine the SSADH activity of germinated Tartary buckwheat.

#### 2.6.3. Determination of Enzymatic Reaction Kinetics Parameters of SSADH

The concentration of NAD^+^ was fixed at 2 mmol/L, the concentration of SSA in the enzyme reaction system was changed, and the SSADH activity of germinated Tartary buckwheat was determined. A concentration of 0.05–0.3 mmol/L SSA was selected to plot the kinetic curve. According to the Lineweaver–Burk double reciprocal plot method, the Km and Vmax of germinated Tartary buckwheat SSADH to substrate SSA were obtained.

The concentration of SSA was fixed at 0.2 mmol/L, and the concentration of NAD ^+^ in the enzyme reaction system was changed to determine the SSADH activity of germinated Tartary buckwheat. The concentration of 0.2–1.2 mmol/L NAD^+^ was selected to plot the kinetic curve. According to the Lineweaver–Burk double reciprocal plot method, the K_m_ and V_max_ of germinated Tartary buckwheat SSADH to coenzyme NAD^+^ were obtained.

### 2.7. Determination Indicators and Methods

#### 2.7.1. Determination of Protein Content

Using bovine serum albumin as the standard, the Coomassie brilliant blue G-250 method was used to determine the protein content [27].

#### 2.7.2. Determination of SSADH Activity

According to Bang et al.’s [28] enzyme activity determination method (with slight modifications), the system included 0.2 mmol/L SSA, 2 mmol/L NAD^+^, 1 mmol/L EDTA, 20 mmol/L β-mercaptoethanol, 1 mmol/L PMSF, and 16 μL crude enzyme solution, supplemented with 1/15 mol/L PBS buffer (pH 8.6) to 200 μL. At 30 °C, the change in absorbance value was measured using a microplate reader (SPECTRA MAX 190 Microplate reader, Meigu Instrument Shanghai Co., Ltd., Shanghai, China) at 340 nm within 10 min, and the enzyme activity was calculated according to the change in the absorbance value. SSADH enzyme activity definition: In the reaction system, the amount of enzyme required to generate 1 nmol NADH ($\sum_{340}^{NADH}$ = 6.22 × 103 M^−1^·cm^−1^) in 1 min was defined as an activity unit (*U*). The enzyme activity calculation formula is as follows:

$$U=\Delta OD\times \frac{1}{t}\times \frac{10^{6}}{\sum_{340}^{NADH}}\times V\times \frac{1}{v}\left(nmol/\min\right)$$

In the formula, Δ*OD*—absorbance increase, *t*—reaction time (min), $\sum_{340}^{NADH}$—molar extinction coefficient of NADH, *V*—total reaction volume (mL), *v*—crude enzyme liquid volume (mL).

### 2.8. Statistical Analysis

The experiment was repeated three times, and the results were expressed as $\;\bar{x}\pm s$. An analysis of variance and correlation analysis were performed using SPSS (version 16.0, Inc., Chicago, IL, USA) software. *p* < 0.05 indicated significant differences, and *p* < 0.01 indicated extremely significant differences. Origin Pro 9.1 64 Bit, Design-Expert. V8.0.6, and Excel 2010 were used to draw curves.

## 3. Results and Discussion

### 3.1. (NH_4_)_2_SO_4_ Fractional Precipitation

#### 3.1.1. Determination of (NH_4_)_2_SO_4_ Saturation

**Figure 1:**
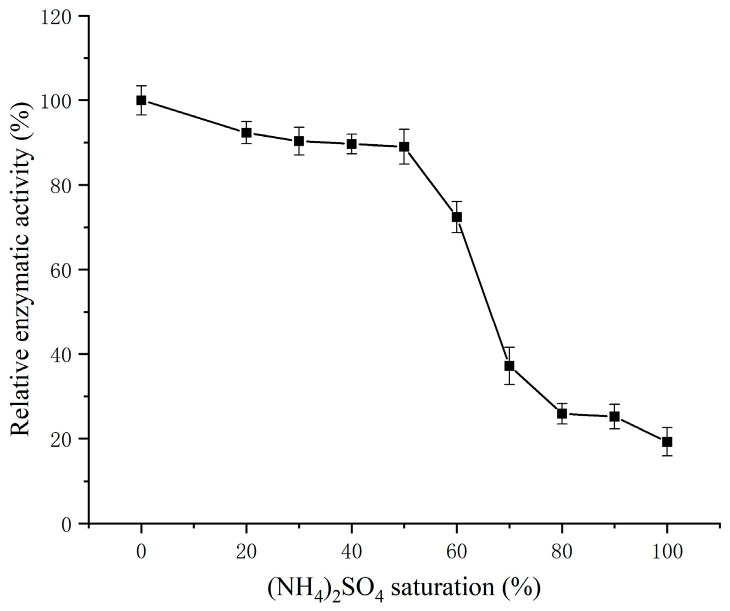
Graded sedimentation of (NH_4_)_2_SO_4_ from the SSADH of germinated Tartary buckwheat.

The SSADH in germinated Tartary buckwheat extract was precipitated using varying saturation levels of (NH_4_)_2_SO_4_, and the residual enzyme activity of SSADH in the supernatant was determined following centrifugation. The corresponding results are illustrated in Figure 1. The SSADH activity in the supernatant significantly decreased as the saturation of (NH_4_)_2_SO_4_ increased from 50% to 80%, indicating that the precipitation of SSADH was most effective at between 50% and 80% (NH_4_)_2_SO_4_ saturation. Therefore, (NH_4_)_2_SO_4_ in a saturation range of 50% to 80% was selected for precipitating the SSADH extract from germinated Tartary buckwheat.

#### 3.1.2. (NH_4_)_2_SO_4_ Fractional Precipitation Results

The enzyme activity and protein content of germinated buckwheat were determined by analyzing the samples collected from the enzyme solution during SSADH extraction and (NH_4_)_2_SO_4_ gradient precipitation. Subsequently, specific activity, purification times, and enzyme activity recovery were calculated for each step. The results are presented in Table 2. After (NH_4_)_2_SO_4_ fractional precipitation, the purification fold of germinated Tartary buckwheat SSADH increased by 3.18-fold, resulting in a specific activity of 84.33 × 10^3^ nmol·min^−1^·mg^−1^, with an enzyme activity recovery rate of 63.76%.

### 3.2. Effect of Temperature on The Determination of SSADH Activity in Germinated Tartary Buckwheat

**Figure 2:**
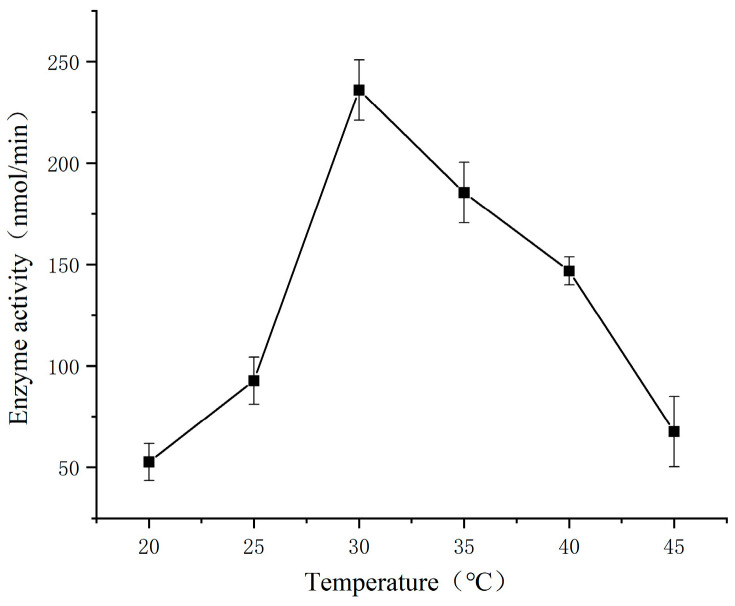
Effect of temperature on the SSADH activity of germinated Tartary buckwheat.

The results of the correlational analysis are presented in Figure 2. At a concentration of 0.2 mmol/L SSA and pH 8.1, the SSADH activity of germinated Tartary buckwheat exhibited a gradual increase with increasing temperature, reaching a maximum of 236.03 nmol/min at 30 °C. Subsequently, as the temperature continued to rise, the SSADH activity gradually declined. The phenomenon can be ascribed to the diminished activity of individual molecules and enzymes within the reaction system at lower temperatures during enzymatic catalysis. As the temperature gradually rises, both enzyme activity and overall molecular activity escalate, accelerating the enzymatic reaction. However, surpassing a certain temperature range results in a rapid decline in reaction rate due to protein denaturation, thereby deactivating the enzyme.

### 3.3. Effect of pH on the Determination of SSADH Activity in Germinated Tartary Buckwheat

**Figure 3:**
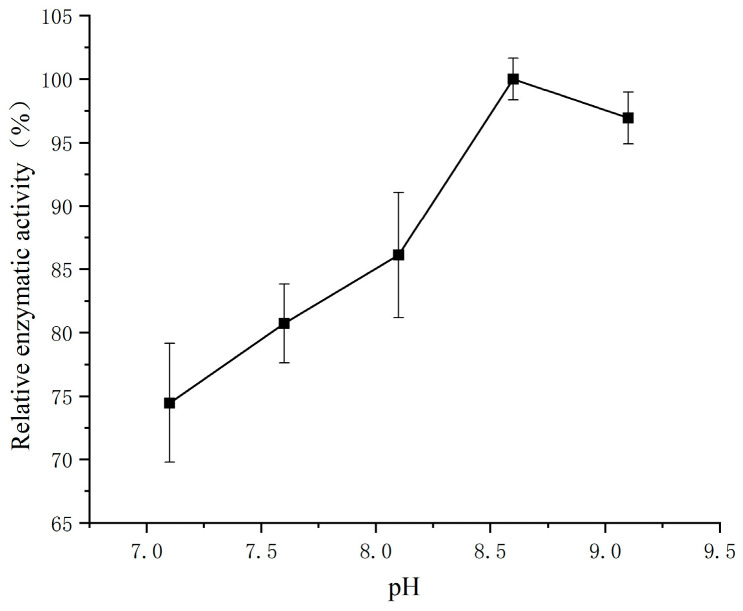
Effects of different pH values on the SSADH activity of germinated Tartary buckwheat.

As shown in Figure 3, at a concentration of 0.2 mmol/L and a temperature of 30 °C, the SSADH activity of germinated Tartary buckwheat exhibited an increasing trend with rising pH until it reached its peak value of 275.12 nmol/min at pH 8.6. Subsequently, further increases in pH led to a decline in SSADH activity due to the influence of reaction medium pH on the enzymatic reactions, which exhibited optimal catalytic efficiency within specific pH ranges. Deviation from this optimum range results in reduced enzyme activity, while excessive acidity or alkalinity can render the enzyme completely inactive. The optimal reaction pH of the subunit of human SSADH was determined to be 8.5, while certain properties of purified barley SSADH exhibited similarities with those found in mammals. Notably, the optimum pH range for purified barley SSADH was observed to be between 9.0 and 10.0, suggesting that there are variations in the optimal pH among different grains [29].

### 3.4. Effect of Substrate SSA Concentration on the SSADH Activity of Germinated Tartary Buckwheat

**Figure 4:**
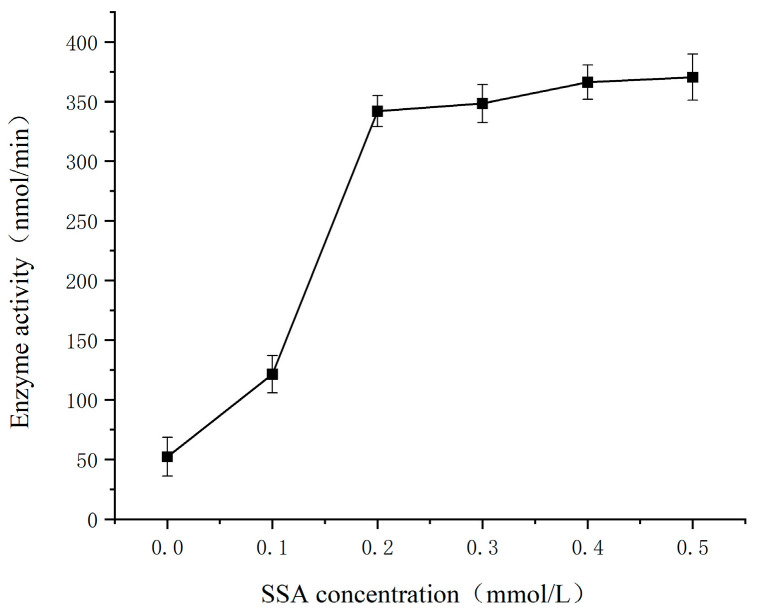
Effect of SSA concentration on SSADH determination of enzyme activity of germinated Tartary buckwheat.

As shown in Figure 4, SSADH activity exhibited a significant increase at 30 °C and pH 8.6 within a SSA concentration range of 0–0.2 mmol/L, followed by a gradual plateauing effect within a SSA concentration range of 0.2–0.5 mmol/L. The phenomenon can be attributed to the dependence of enzymatic reaction rate on substrate concentration in an enzymatic reaction system with constant enzyme concentration, reaction temperature, and pH. At low substrate concentrations, the enzymatic reaction is accelerated by increasing substrate concentration, exhibiting a proportional relationship between reaction rate and substrate concentration. However, as the substrate concentration increases further, the rate of the increase in reaction rate is diminished. If the substrate concentration continues to rise beyond this point, the maximum limit (V_max_) of the reaction rate will be approached.

### 3.5. Determination Conditions of SSADH Enzyme Activity Box–Behnken Experimental Model Analysis of Variance

#### 3.5.1. Response Surface Experimental Design and Results

The Box–Behnken design was used, and response surface analysis tests were conducted using temperature, pH value, and SSA concentration as independent variables, while enzyme activity was considered as the response variable. The obtained results are presented in Table 3. Multiple regression fitting of the data was performed using Design Expert V8.0.6 software. The quadratic polynomial equation describing the relationship between SSADH enzyme activity and the independent variables (temperature, pH value, and SSA concentration) in this study is provided in Equation (1) below:

*Y*= 313.69 + 20.29*A* + 16.54*B* + 76.58*C* + 19.72*AB* + 7.88*AC* − 2.83*BC* − 93.99*A*^2^ − 66.81*B*^2^ − 44.60*C*^2^ (1)

#### 3.5.2. Regression Model and Variance Analysis

**Figure 5:**
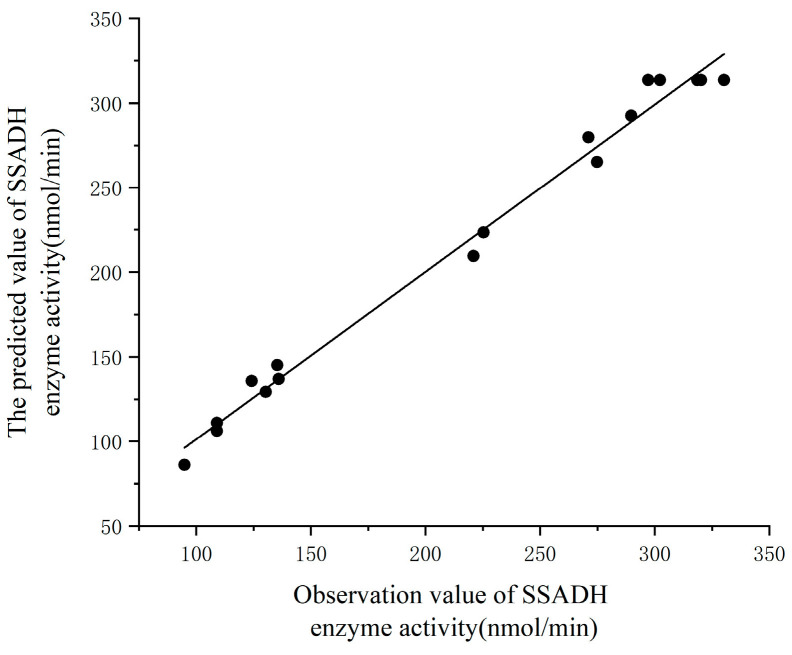
Correlation between predicted and experimental results regarding SSADH activity content.

The analysis of variance (Table 4) revealed that the model was highly significant (*p* < 0.001), indicating its strong statistical significance. Additionally, the model also possessed no significant lack of fit (*p* > 0.05). The regression equation demonstrated a determination coefficient of 0.9892 and a coefficient of variation of 6.45. Moreover, the value of the adjusted determination coefficient (R^2^_adj_ = 0.9752) suggested that this model effectively analyzed and predicted the relationship between factors and response values, explaining approximately 97.52% of their variations. Furthermore, the comparison between the predicted values of SSADH activity (Figure 5) showed a very significant correlation. Moreover, with an SNR value exceeding 21.187 compared to a threshold of 4, it indicated that this model exhibited excellent fit with experimental data and possessed minimal experimental error, thus enabling an effective analysis of the relationship between germinated Tartary buckwheat’s SSADH activity and its respective variables.

### 3.6. Response Surface Analysis and Optimization of SSADH Activity Determination Conditions of Germinated Tartary Buckwheat

**Figure 6:**
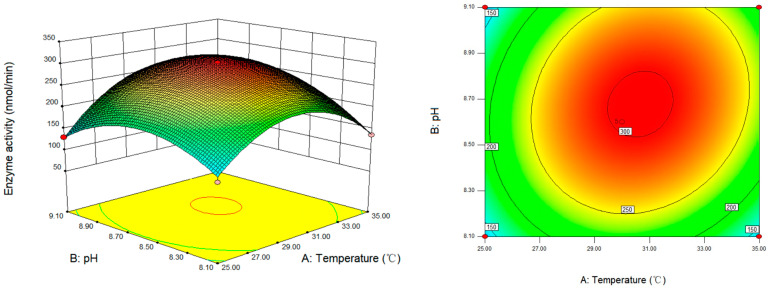
Response surface and contour map of the interaction between temperature and pH on SSADH activity of germinated Tartary buckwheat (SSA 0.2 mmol/L).

According to regression Equation (1), a response surface diagram and contour map were constructed to analyze the relationship between various factors and the activity of SSADH in germinated Tartary buckwheat. Figure 6 illustrates the impact of temperature and pH on determining SSADH activity in germinated Tartary buckwheat with a fixed SSA concentration of 0.2 mmol/L. The linear term (*p* < 0.05) for temperature and pH significantly influenced the determination of SSADH enzyme activity, while the quadratic term (*p* < 0.001) had a highly significant effect. Additionally, there was a significant interaction between temperature and pH (*p* < 0.05). Under specific pH conditions, SSADH enzyme activity initially increased with increasing temperature before reaching its peak at 30.79 °C, indicating that temperature played a role in affecting SSADH enzyme activity, with optimal determination occurring at 30.79 °C. At constant temperature, an increase in pH from 8.1 to 8.66 led to a rapid increase in SSADH activity; however, further increases in pH resulted in decreased enzymatic activity levels, suggesting that a pH value of 8.66 facilitated the accurate determination of SSADH activity in germinated Tartary buckwheat.

**Figure 7:**
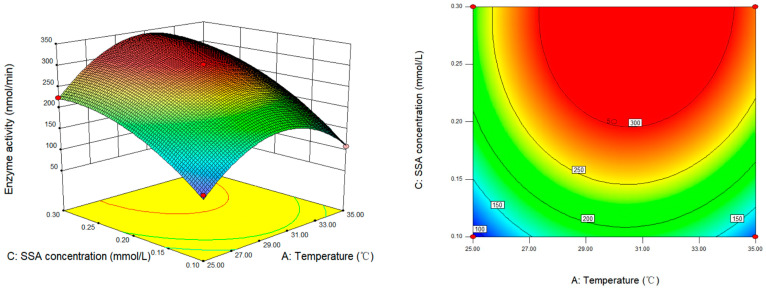
Response surface and contour map of the interaction between temperature and SSA concentration on SSADH enzyme activity of germinated Tartary buckwheat (pH 8.6).

The effects of temperature and SSA concentration on the determination of SSADH activity in germinated Tartary buckwheat are presented in Figure 7. Our model and variance analyses revealed that the interaction between temperature and SSA concentration was not statistically significant (*p* > 0.05). The activity of SSADH exhibited a positive correlation with increasing SSA concentrations under specific temperature conditions, reaching its maximum at 0.29 mmol/L SSA. Subsequent elevations in SSA concentration did not exert a significant impact on the SSADH enzyme activity.

**Figure 8:**
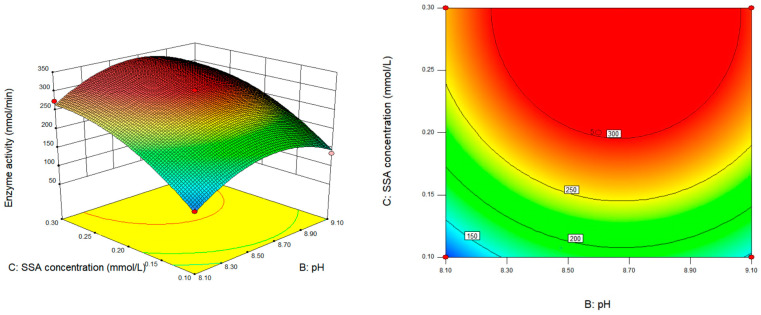
Response surface and contour map of the interaction between pH and SSA concentration on SSADH activity of germinated Tartary buckwheat (30 °C).

The interaction between pH and SSA concentration was not found to be significant based on Figure 8 (*p* > 0.05). SSADH activity increased with increases in pH and SSA concentration. It should be noted that excessively high pH levels were found to have a negative impact on the accurate determination of SSADH activity in germinated Tartary buckwheat.

In summary, based on the response surface test results, the optimal determination conditions for predicting SSADH enzyme activity in germinated Tartary buckwheat using a regression model were as follows: temperature—330.79 °C, pH—8.66, and SSA concentration—0.29 mmol/L. Under these conditions, the predicted SSADH enzyme activity was 349.60 nmol/min. To assess the reliability of the model, a verification test was conducted. Considering practical feasibility, the modified determination conditions were as follows: temperature—30.8 °C, pH—8.7, and SSA concentration—0.3 mmol/L. Under these revised conditions, the observed SSADH enzyme activity in germinated Tartary buckwheat was found to be within a range of 346 ± 9.61 nmol/min compared to theoretical values with minimal relative error. This finding confirms that our model is reliable and capable of accurately predicting SSADH enzyme activity in germinated Tartary buckwheat.

### 3.7. Thermal Stability and pH Stability of Germinated Tartary Buckwheat SSADH

**Figure 9:**
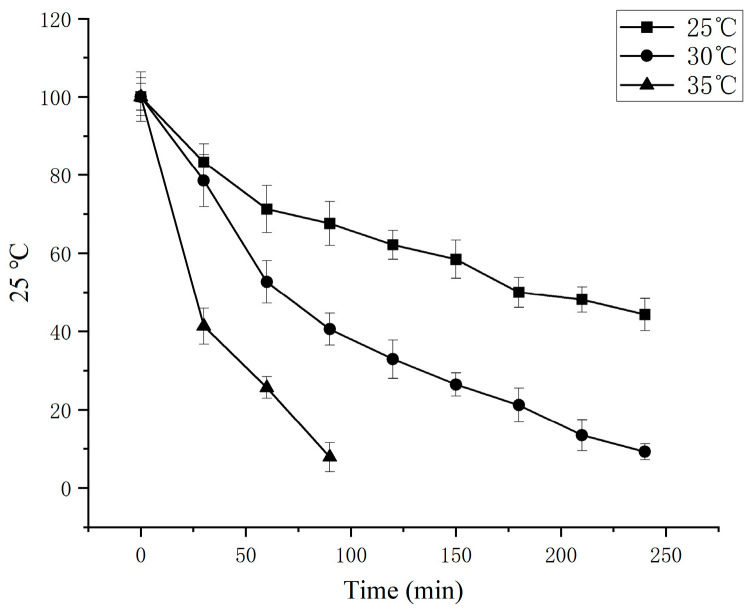
Thermal stability of germinated Tartary buckwheat.

**Figure 10:**
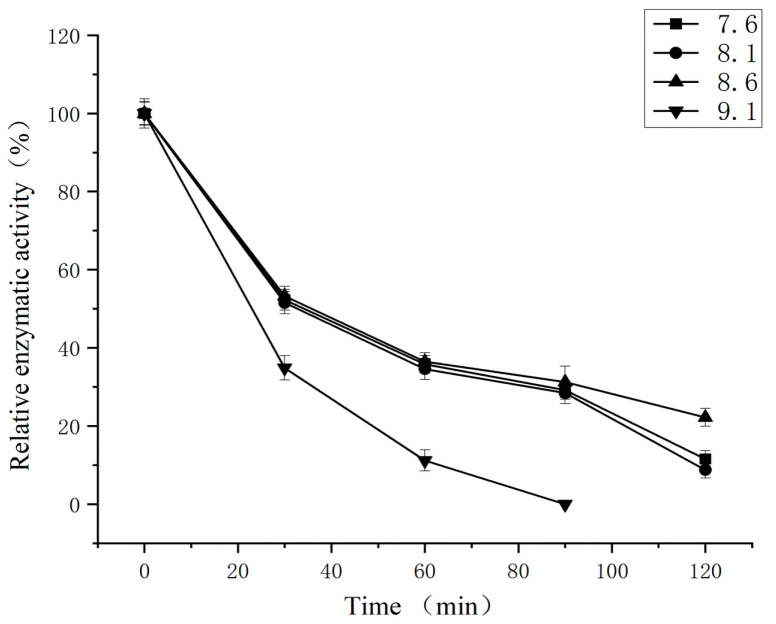
pH stability of germinated Tartary buckwheat.

The thermal stability of germinated buckwheat SSADH was investigated at temperatures of 25, 30, and 35 °C. As depicted in Figure 9, the highest thermal stability was observed at 25 °C. The enzyme half-life of SSADH at 25 °C was 180 min, which was better than 64.5 min at 30 °C and 25.6 min at 35 °C. The catalytic efficiency of the enzymatic reaction medium is significantly influenced by its pH level due to its ability to modify the dissociation degree of amino acid groups within the enzyme’s active center [30]. Therefore, an investigation into the pH stability of germinated buckwheat SSADH was conducted under conditions with pH values ranging from 7.6 to 9.1 (Figure 10). The results indicated comparable levels of stability for pH values of 7.6, 8.1, and 8.6, with respective half-lives of 34.2 min, 32.7 min, and 36 min. At a pH value of 9.1, the enzyme exhibited poor stability, with a half-life of 23 min.

### 3.8. Effects of Metal Ions on SSADH Activity in Germinated Tartary Buckwheat

**Figure 11:**
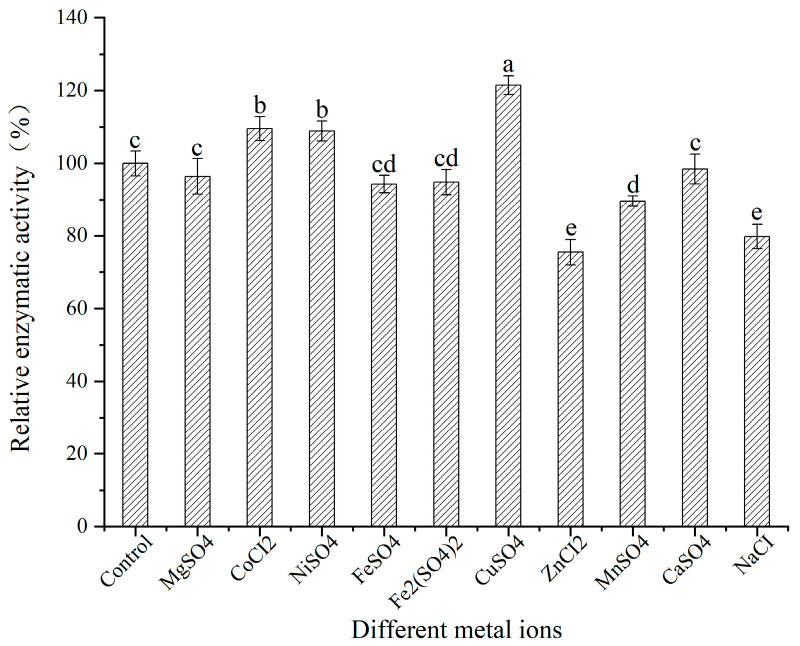
Effects of different metal ions on the SSADH activity of germinated Tartary buckwheat Different Superscript letters (a, b, c, d, e) indicate statistically significant differences among groups.

The impact of various metal ions on the activity of SSADH in germinated Tartary buckwheat was investigated, with the enzyme reaction without the addition of metal ions serving as a control group. Cu^2+^, Co^2+^, and Ni^2+^ demonstrated an activating effect on SSADH activity in germinated Tartary buckwheat (Figure 11), with Cu^2+^ exhibiting the most significant impact (*p* < 0.05), being 1.21 times higher than that of the control group. Conversely, Zn^2+^, Mn^2+^, and Na^+^ inhibited SSADH activity in germinated Tartary buckwheat, with Zn^2+^ exerting the strongest inhibitory effect (*p* < 0.05). Mg^2+^, Fe^2+^, Fe^3+^, and Ca^2+^ had no discernible influence on SSADH activity, showing no statistically significant differences among them (*p* > 0.05). Teisseire et al. [31] also demonstrated that some metal ions can enhance the activities of catalase and certain peroxidases, which is consistent with our results.

### 3.9. Enzymatic Kinetic Parameters of SSADH on Substrate SSA and Coenzyme NAD^+^ in Germinated Tartary Buckwheat

**Figure 12:**
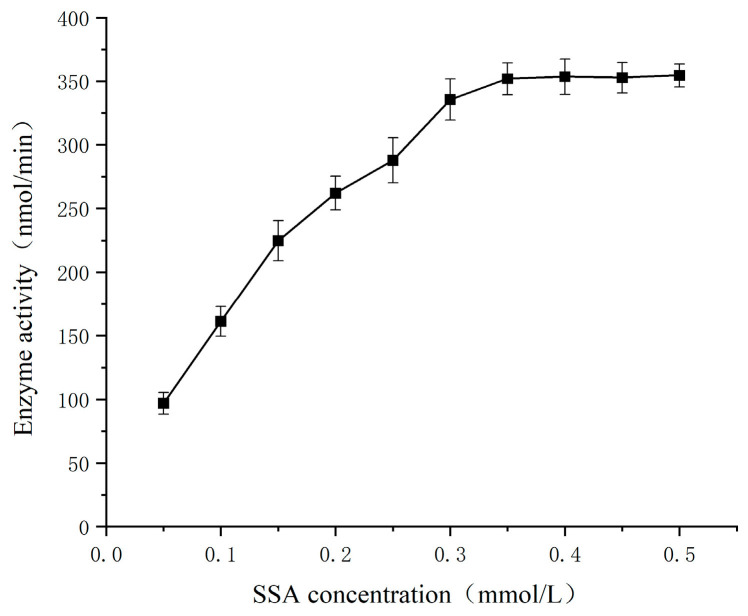
Effect of SSA concentration on the SSADH activity of germinated Tartary buckwheat.

**Figure 13:**
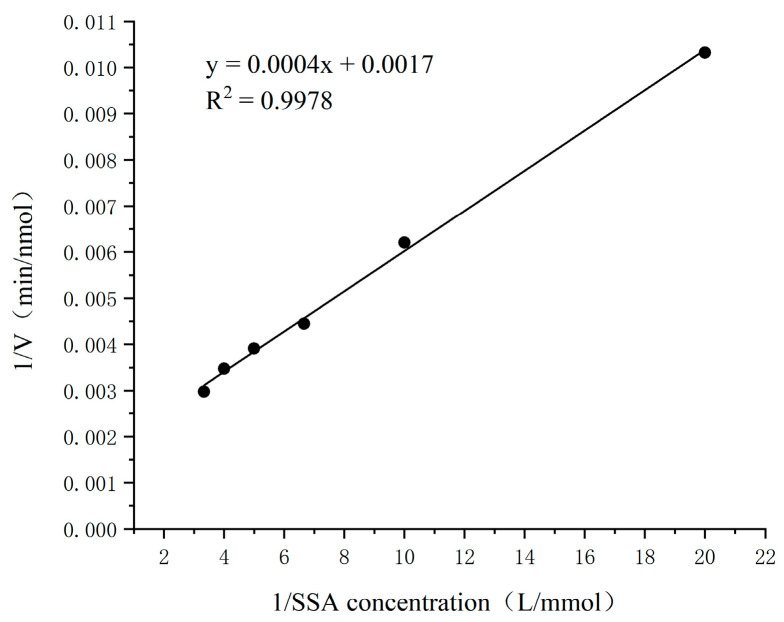
Lineweaver–Burk dynamics chart of the SSADH of germinated Tartary buckwheat (SSA).

**Figure 14:**
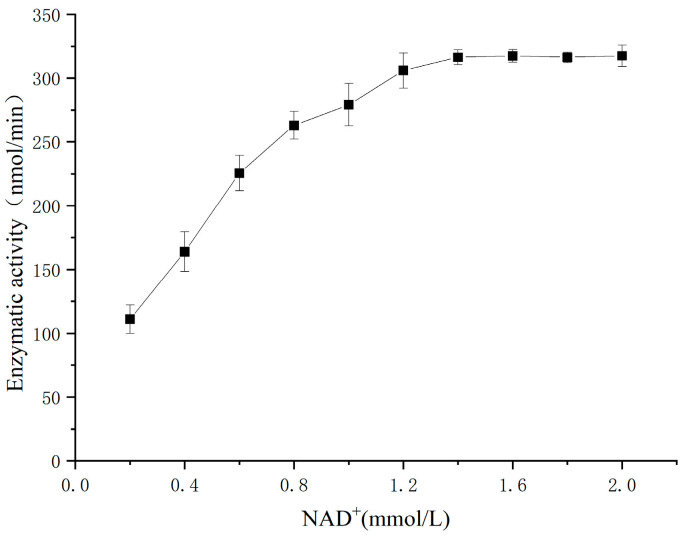
Effect of coenzyme NAD^+^ concentration on the SSADH activity of germinated Tartary buckwheat.

**Figure 15:**
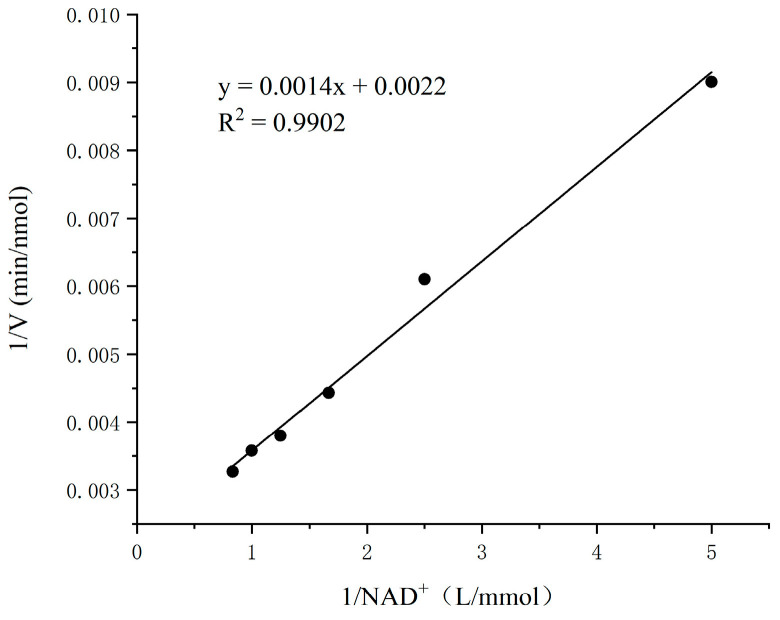
Lineweaver–Burk dynamics chart of SSADH of germinated Tartary buckwheat (NAD^+^).

The kinetic parameters of germinated Tartary buckwheat SSADH were investigated in a 1/15 mmol/L PBS buffer (pH 8.6) at 30 °C using SSA as the substrate and 2 mmol/L NAD^+^ as the cofactor. The results are presented in Figure 12. The enzymatic reaction rate of SSADH gradually approached its maximum when the concentration of SSA ranged from 0.05 to 0.3 mmol/L. Within the range of 0.3–0.5 mmol/L, the reaction rate remained relatively constant and reached its peak level. To determine the SSADH enzyme reaction rate of germinated Tartary buckwheat, experiments were conducted within SSA concentrations ranging from 0.05 to 0.3 mmol/L, and a Lineweaver–Burk kinetic diagram was constructed (Figure 13). The calculated K_m_ and V_max_ values for substrate SSA were found to be approximately equal to 0.2353 mmol/L and approximately equal to 583.2353 nmol/min, respectively. Subsequently, by maintaining a fixed concentration of SSA at 0.2 mmol/L and manipulating the concentration of NAD^+^, it was observed that the coenzyme NAD^+^ exerted a discernible impact on SSDH activity in germinated Tartary buckwheat (Figure 14). When the NAD^+^ concentration was gradually increased in the range of 0.2 to 1.2 mmol/L, the enzymatic reaction rate gradually reached a maximum. The reaction rate of the SSADH enzyme in sprouting Tartary buckwheat was measured within this range, and the Lineweaver–Burk kinetic diagram was drawn (Figure 15). The K_m_ and V_max_ of SSADH to the coenzyme NAD^+^ in germinate Tartary buckwheat were 0.6364 mmol/L and 454.5455 nmol/min, respectively. The catalytic activity of SSADH has been demonstrated to be closely associated with its origin. For instance, SSADH from the Ynel class derived from Bacillus subtilis exhibited negligible disparity in catalytic activity between the two coenzyme factors [32]. Conversely, SSADH from Escherichia coli belonging to the GabD class displayed a 20-fold higher catalytic activity when utilizing NADP^+^ compared to when utilizing NAD^+^ [33].

## 4. Conclusions

In this study, the assay conditions for the SSADH activity of germinating Tartary buckwheat were optimized using the Box–Behnken experimental design, and the enzymatic properties of SSADH were further investigated. The optimal conditions for determining SSADH activity in germinated Tartary buckwheat were found to be as follows: temperature—30.8 °C, pH—8.7, and SSA concentration—0.3 mmol/L. Under these conditions, the measured SSADH activity in germinated Tartary buckwheat was determined to be 346 ± 9.61 nmol/min. This study revealed a significant correlation between the activity of SSADH and pH, with a decrease observed at pH levels higher than 9. Furthermore, the activity of SSADH is distinctly influenced by a variety of metal ions. The findings presented in this study provide a theoretical framework for understanding the mechanism underlying GABA enrichment in germinating buckwheat while also providing essential technical assistance for the future commercial development of GABA-rich Tartary buckwheat foods in China.

## Figures and Tables

**Table 1:** Factors and levels of RSM analysis.

Variable	Number	Experimental Level		
		−1	0	1
temperature (°C)	A	25	30	35
pH value	B	8.1	8.6	9.1
SSA potency (mmol/L)	C	0.1	0.2	0.3

**Table 2:** Graded sedimentation of (NH_4_)_2_SO_4_ from the SSADH of germinated Tartary buckwheat.

Purification Procedure	Total Protein (mg)	Total Activity (nmol·min^−1^)	Specific Activity (nmol·min^−1^·mg^−1^)	Purification Multiple	Live Enzyme Recovery Rate (%)
Tartary buckwheat SSADH extract	28.56	756.56 × 10^3^	26,491.60	1	100
(NH_4_)_2_SO_4_ fractional precipitation of crude enzyme solution	5.72	482.36 × 10^3^	84,328.67	3.18	63.76

**Table 3:** Box–Behnken design and experiment date for predicted and actual content.

Number	Factors and Levels			Enzymatic Activity	
	*A*	*B*	*C*	Actual Value	Predicted Value
1	−1	−1	0	124.26	135.78
2	1	−1	0	136.00	136.91
3	−1	1	0	130.33	129.42
4	1	1	0	220.96	209.44
5	−1	0	−1	94.87	86.11
6	1	0	−1	109.08	110.93
7	−1	0	1	225.36	223.51
8	1	0	1	271.07	279.83
9	0	−1	−1	109.08	106.32
10	0	1	−1	135.41	145.08
11	0	−1	1	274.81	265.14
12	0	1	1	289.80	292.56
13	0	0	0	302.34	313.69
14	0	0	0	318.56	313.69
15	0	0	0	320.15	313.69
16	0	0	0	330.26	313.69
17	0	0	0	297.12	313.69

**Table 4:** Analysis of variance (ANOVA) for the regression equation.

Source of Variance	Quadratic Sum	Free Degree	Mean Square	F-Value	*p*-Value	Conspicuousness
model	1.253 × 10^5^	9	13,922.42	70.98	<0.0001	**
*A*—temperature	3292.26	1	3292.26	16.78	0.0046	**
*B*-pH	2189.57	1	2189.57	11.16	0.0124	*
*C*—SSA potency	46,909.85	1	46,909.85	239.16	<0.0001	**
*AB*	1555.91	1	1555.91	7.93	0.0259	*
*AC*	248.06	1	248.06	1.26	0.2978	
*BC*	32.15	1	32.15	0.16	0.6977	
*A* ^2^	37,195.70	1	37,195.70	189.63	<0.0001	**
*B* ^2^	18,793.58	1	18,793.58	95.81	<0.0001	**
*C* ^2^	8376.07	1	8376.07	42.70	0.0003	**
residual	1373.03	7	196.15			
lack of fit	629.63	3	209.88	1.13	0.4373	
pure error	743.40	4	185.85			
total variation	1.267 × 10^5^	16				
		R^2^ = 0.9892	R^2^_adj_ = 0.9752	R_SN_ = 21.187	C.V = 6.45%	

Note: * the difference is significant (*p* < 0.05); ** the difference is highly significant (*p* < 0.01).

## Data Availability

The data presented in this study are available upon request from the corresponding author.
